# Growth-Phase Sterigmatocystin Formation on Lactose Is Mediated via Low Specific Growth Rates in *Aspergillus nidulans*

**DOI:** 10.3390/toxins8120354

**Published:** 2016-11-28

**Authors:** Zoltán Németh, Ákos P. Molnár, Balázs Fejes, Levente Novák, Levente Karaffa, Nancy P. Keller, Erzsébet Fekete

**Affiliations:** 1 Department of Biochemical Engineering, Faculty of Science and Technology, University of Debrecen, Egyetem tér 1, Debrecen H-4032, Hungary; nemeth.zoltan@science.unideb.hu (Z.N.); molnar.akos@science.unideb.hu (A.P.M.); fejes.balazs91@gmail.com (B.F.); karaffa.levente@science.unideb.hu (L.K.); 2 Department of Physical Chemistry, Faculty of Science and Technology, University of Debrecen, Egyetem tér 1, Debrecen H-4032, Hungary; novak.levente@science.unideb.hu; 3 Department of Medical Microbiology and Immunology, University of Wisconsin, Madison, WI 53706, USA; npkeller@wisc.edu; 4 Department of Bacteriology, University of Wisconsin, Madison, WI 53706, USA

**Keywords:** *Aspergillus nidulans*, sterigmatocystin, lactose, d-glucose, CreA, growth rate

## Abstract

Seed contamination with polyketide mycotoxins such as sterigmatocystin (ST) produced by *Aspergilli* is a worldwide issue. The ST biosynthetic pathway is well-characterized in *A. nidulans*, but regulatory aspects related to the carbon source are still enigmatic. This is particularly true for lactose, inasmuch as some ST production mutant strains still synthesize ST on lactose but not on other carbon substrates. Here, kinetic data revealed that on d-glucose, ST forms only after the sugar is depleted from the medium, while on lactose, ST appears when most of the carbon source is still available. Biomass-specified ST production on lactose was significantly higher than on d-glucose, suggesting that ST formation may either be mediated by a carbon catabolite regulatory mechanism, or induced by low specific growth rates attainable on lactose. These hypotheses were tested by d-glucose limited chemostat-type continuous fermentations. No ST formed at a high growth rate, while a low growth rate led to the formation of 0.4 mg·L^−1^ ST. Similar results were obtained with a CreA mutant strain. We concluded that low specific growth rates may be the primary cause of mid-growth ST formation on lactose in *A. nidulans*, and that carbon utilization rates likely play a general regulatory role during biosynthesis.

## 1. Introduction

Aflatoxins (AF) are among the most carcinogenic natural substances known to date and the most important mycotoxins. They are produced by a large diversity of ascomycetous fungal species, mainly from the genus *Aspergillus* [1]. These fungi can contaminate cereal crops and other staple commodities before harvest or during storage, leading to huge economic losses worldwide, and occasional famine in tropical countries [2]. Upon exposure, the toxins can cause acute hepatic failure in humans and animals [3].

Sterigmatocystin (ST) is structurally similar to AF—both are members of a large and diverse class of compounds known as polyketides [4]—but is less potent [5]. In fact, ST is the penultimate intermediate in the biosynthesis of AF in *A. flavus*; however, in several other fungi including the model fungus *A. nidulans*, it is the end product of the corresponding pathway [6], lacking the last two genes present in *A. flavus* and other AF producing fungi [7].

In addition to being a model system for biochemical and genetic research in multi-cellular fungi [8], *A. nidulans* is also the industrial producer of the antifungal agent echinocandin B which inhibits the synthesis of glucan, a major component of the fungal cell wall [9]. It has also been used to investigate the biosynthesis of the beta-lactam antibiotic penicillin [10]. Furthermore, it has been employed as a heterologous host for the industrial or pilot-scale production of other prominent natural substances such as the cholesterol lowering drug lovastatin [11] and the immunosuppressive agent mycophenolic acid [12]. In contrast to standard genetic practices where fungal strains are typically cultivated on agar-solidified media on small plastic plates, these processes are carried out as aerobic, liquid (submerged) fermentations with relatively high mycelial density. Amounts do matter: the formation of ST as a contaminant during such fermentations could eventually turn out to be an immense safety issue [13] leading to efforts by industry to find *A. nidulans* strains blocked in ST production [14].

While the AF/ST biosynthetic pathway is well-characterized in *A. nidulans* with all the structural genes and intermediates described, many of the regulatory aspects, including those related to the carbon source available for the fungus, are still enigmatic [15]. This is particularly true for the heterodisaccharide lactose (milk sugar; 1,4-*O*-β-d-galactopyranosyl-d-glucose), the main carbohydrate in cheese whey, which is traditionally considered a cheap and abundant industrial growth substrate for micro-organisms, fungi in particular. The “lactose effect” can be summarized as the ability of some *A. nidulans* ST production mutants to still synthesize ST on lactose. For example, certain *A. nidulans* oxylipin (endogenous fatty acid-derived molecules) mutants unable to produce ST on regular glucose supplemented medium were able to produce ST on lactose-supplemented medium [16]. In addition, contributions of mitochondrial and/or peroxisomal beta-oxidation to ST formation are essential on all carbon sources but lactose, as disruption of the mitochondrial enoyl-CoA hydratase and/or the d-bifunctional protein homolog in the peroxisomal beta-oxidation pathway did not influence ST levels on lactose [15].

In this study, a mechanism for the aforementioned lactose effect is proposed. We will provide evidence that low specific growth rates typical on lactose are the primary cause of sustained, mid-growth ST formation in *A. nidulans*, and that carbon assimilation rates likely play a general regulatory role during biosynthesis.

## 2. Results

### 2.1. Verification of the Experimental Strategy

**Figure 1:**
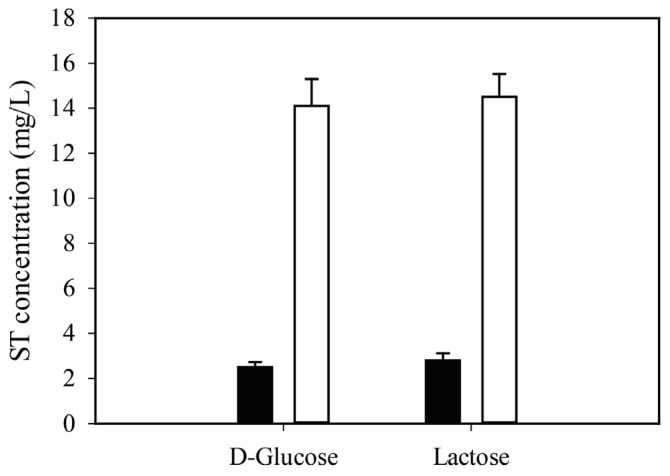
Maximal sterigmatocystin (ST) production of the *A. nidulans* wild-type strain RDIT 9.32 as a function of the carbon source. d-glucose and lactose indicates minimal media initially containing 15 g/L sole carbon substrate. Black and white columns indicate liquid and agar-solidified cultures, respectively. Data presented here are means of three independent experiments (biological replicates). The variations among experiments were estimated by standard deviations (SDs), indicated by the error bars. ST extraction and quantification protocols are described in details in Section 4.

While the production rates of ST under liquid (=submerged, batch) conditions are four to five times lower than on solidified medium on lactose as well as on d-glucose (Figure 1), an optimized extraction and analytical protocol still allowed us to determine the actual ST concentration in a reproducible way on both carbon sources of interest (see Section 4 for details). The detection limit was 0.1 mg·L^−1^, which is safely over the concentration range *A. nidulans* can produce under submerged conditions, even in minimal medium.

**Figure 2:**
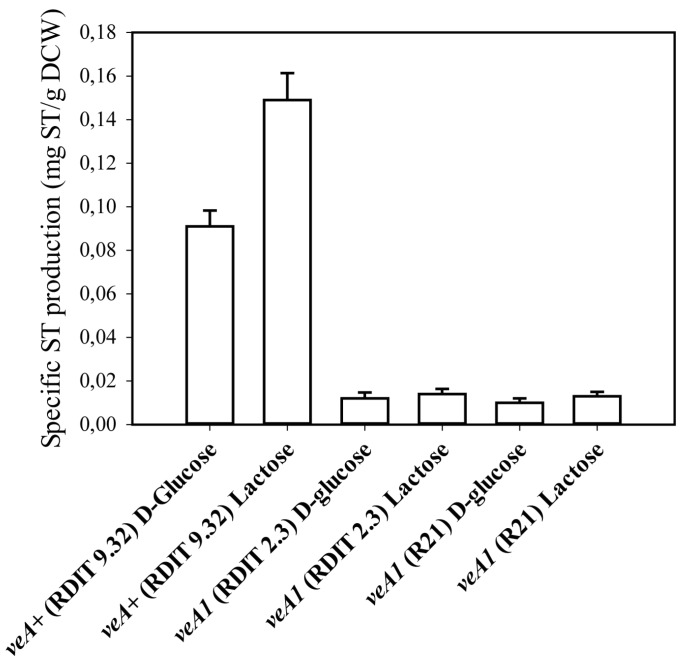
Maximal biomass-specific sterigmatocystin (ST) production of *A. nidulans* strains RDIT 9.32 and RDIT 2.3, carrying the *veA+* (wild-type) allele, as well as of the *A. nidulans* R21 strain carrying the *veA1* (mutant) allele in liquid minimal medium initially containing 15 g/L d-glucose or lactose as sole carbon sources. For additional information on the strains, see Table 3. DCW: dry cell weight.

The velvet gene (*veA*) of *A. nidulans* is involved in the regulation of a variety of cellular processes such as asexual and sexual development as well as secondary metabolism [17]. A single base pair mutation at the full length *veA* start codon called *veA1* was described to impair ST production [18]. To see whether this is also the case under the conditions of this study, two *veA1* mutant strains were tested for ST production and compared to a wild-type (*veA+*) strain (Figure 2 and Table 3). On both carbon sources, biomass-specified ST production was 10–15 times higher in the strain containing the wild-type allele. In fact, maximal ST concentrations in the *veA1* mutant cultures were just slightly above the detection limit of our system. It is to be noted that while maximal ST concentration values (given in mg·L^−1^) were higher on d-glucose than on lactose, biomass-specific (μg_ST_·g_DCW_^−1^) ST production, conversely, was significantly (*p* < 1%) higher on lactose, due to the comparatively reduced growth on this carbon source. Data also revealed that even in a *veA+* (wild-type) background, the ratio of ST produced to biomass formed is low, approximately one to ten-thousand.

**Figure 3:**
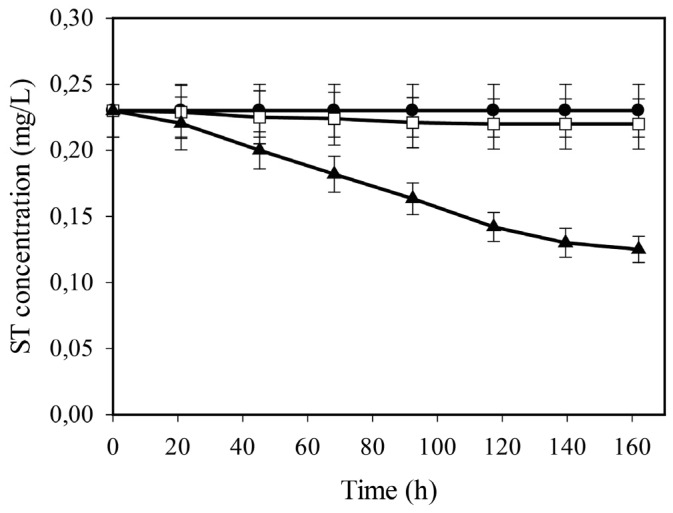
Time-profile of residual sterigmatocystin (ST) concentrations in sterile water (●), in sterile minimal growth medium with d-glucose (**□**) and in *A. nidulans* liquid batch cultures with d-glucose as a sole carbon source (▲).

In agreement with the reported high stability of ST in aqueous solutions at neutral pH [19], its concentration in buffered water remained unchanged even after a one week-long incubation period in a rotary shaker mimicking the physical parameters of the fungal fermentations, and the same happened during incubation in sterilized liquid growth medium under the same conditions (Figure 3). However, micro-organisms including fungi are known to be capable of degrading AF [20,21]. To test whether the biodegradation of ST exists under the conditions of this study, glycerol-pregrown, *veA1*-type (i.e., ST-non-producing) *A. nidulans* cultures were externally supplemented with ST and cultivation continued for up to a week (Figure 3). ST-concentrations did decrease with time at an average rate of 0.14 μg·g_DCW_^−1^·h^−1^. This value showed that while *A. nidulans* does have the potential to degrade this mycotoxin—and thus theoretically, the actual concentration of ST at any time-point of the cultivation is the net resultant of production and degradation—the rate of the latter remained far below that of the former, and thus did not likely affect the general strategy we wished to employ here. We therefore considered the experimental system appropriate for the purposes of this study.

### 2.2. Kinetics of the Wild-Type Sterigmatocystin Production in Batch and Fed-Batch Cultures

**Figure 4:**
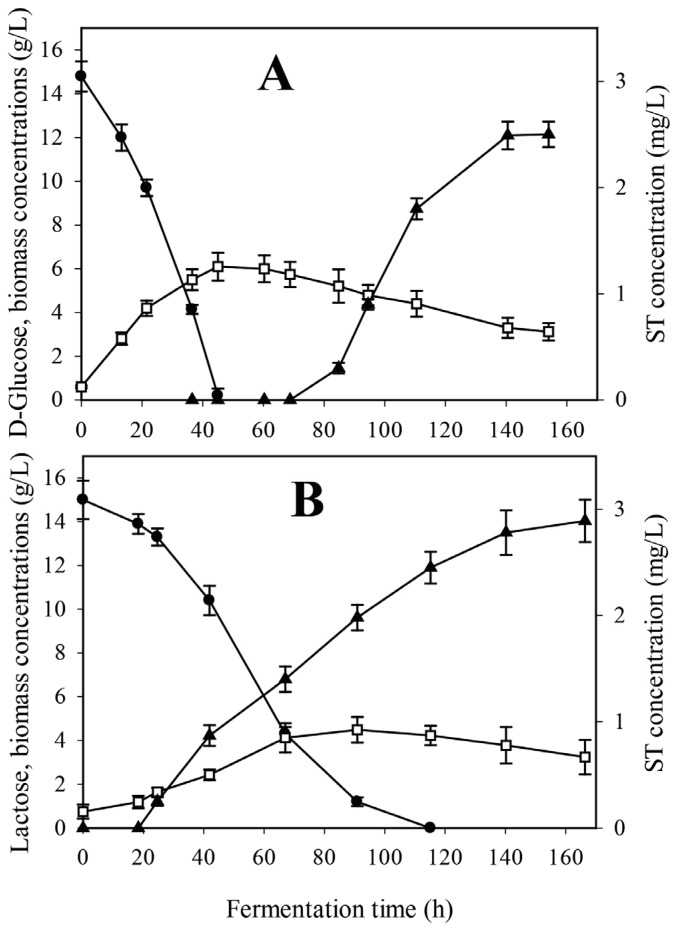
Time-profile of growth (□), residual carbon source concentrations (●) as well as sterigmatocystin (ST) production (▲) in batch fermentations of an *A. nidulans* wild-type strain in minimal media initially containing 15 g/L sole carbon substrate. (**A**) d-glucose; (**B**) lactose. Mycelial inoculum preformed overnight on glycerol and transferred into the bioreactors was used for all fermentations.

In order to analyse the relationship between carbon utilization rate and ST formation in *A. nidulans*, we cultivated the wild-type (*veA*) strain on minimal medium with d-glucose—which is rapidly metabolized—and the slowly assimilated lactose. Time-profiles of ST formation were markedly different: on d-glucose, ST could be detected only after d-glucose was completely depleted from the medium, which happened after approximately 1.5 days of cultivation (Figure 4A), while on lactose, ST appeared already in the growth phase, when the majority of the carbon source was still readily available in the medium (Figure 4B). Although growth stopped as a result of d-glucose depletion, ST production continued unabated for an additional 70 h, ultimately reaching a concentration of 2.5 mg·L^−1^. The final ST titer in lactose medium was somewhat higher (2.8 mg·L^−1^), resulting in a significant difference regarding biomass-specified ST production.

**Figure 5:**
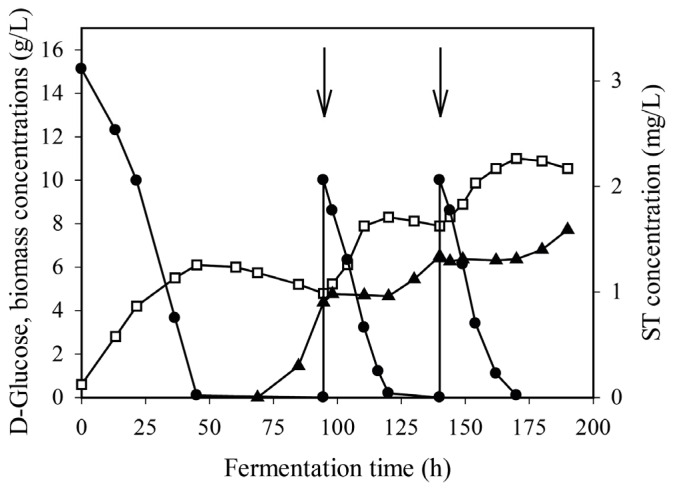
Time profiles of growth (□), residual d-glucose concentrations (●) as well as sterigmatocystin (ST) production (▲) in fed-batch fermentations of an *A. nidulans* wild-type strain in minimal media. Additional d-glucose (indicated by plain arrows) was added at 94 h and 140 h. To increase clarity, the plot gives the mean data of three independent fermentations instead of displaying error bars. The mean standard deviation for the d-glucose concentration was 5% and for the biomass concentration, 10%; the maximum deviations were 7% and 13%, respectively. Inoculation of the bioreactor occured as described above.

The aforementioned kinetic data suggested that ST production does not occur before the complete exhaustion of d-glucose. To investigate whether the reverse of this correlation also holds true—i.e., whether d-glucose inhibits already ongoing ST production—we employed fed-batch cultivations. By the time ST concentration in the wild-type *A. nidulans* culture reached 1 mg·L^−1^ (at approximately 94 h), extra d-glucose was added to the medium. As can be seen (Figure 5), the extra d-glucose mainly went to renewed biomass production, whereas ST-production stopped immediately. The d-glucose was again rapidly exhausted, after which ST production resumed. Essentially identical kinetic patterns were observed upon addition of another d-glucose pulse at 140 h.

### 2.3. Kinetics of Sterigmatocystin Production by A. nidulans in a creA-Negative Background

**Figure 6:**
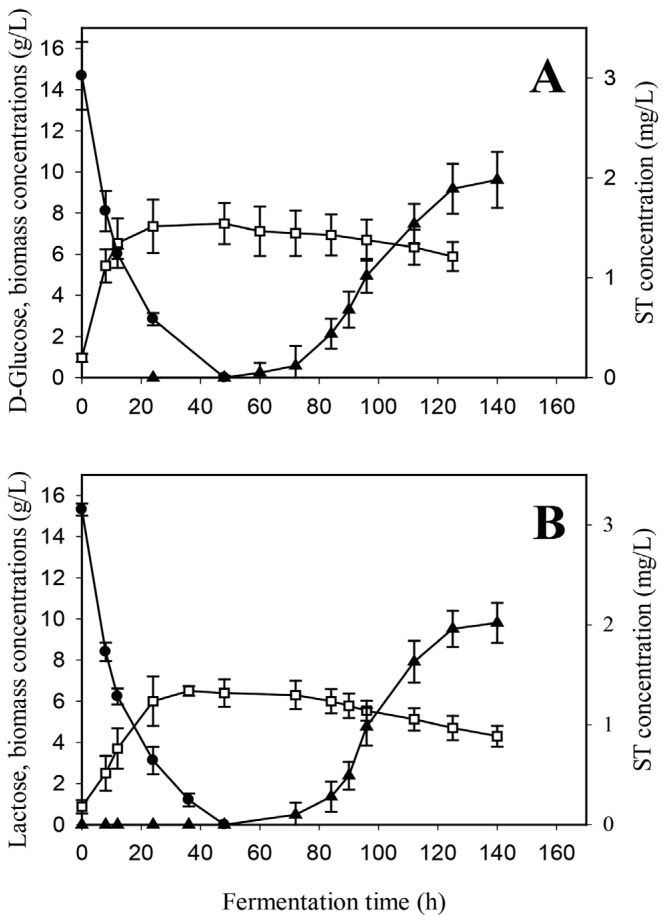
Time profile of growth (□), residual carbon source concentrations (●) as well as sterigmatocystin (ST) production (▲) in batch fermentations of an *A. nidulans* carbon catabolite derepressed CreA mutant strain in minimal media initially containing 15 g/L sole carbon substrate. (**A**) d-glucose; (**B**) lactose. Inoculation of the bioreactor occurred as described above.

The results above suggested that ST-formation in *A. nidulans* may either be mediated by a carbon catabolite regulatory mechanism prominent on d-glucose, or induced by the low specific growth rate typical on lactose and under d-glucose limitation. To test this hypothesis, we repeated the submerged fermentations with a carbon catabolite derepressed mutant strain (*ΔcreA*, *veA+)*. The time courses of d-glucose uptake and biomass formation were similar to those of the wild-type strain, whereas lactose was exhausted more rapidly from the medium relative to the reference strain (Figure 6A,B). In fact, lactose disappeared from the growth medium of the CreA-mutant almost as rapidly as d-glucose did from the wild-type culture, resulting in an unusually high biomass yield (*Y*_x/s_) for this disaccharide (Table 1). Most importantly, ST appeared only at the very late stages of the lactose fermentations, when mycelia were already starting to disintegrate (Figure 6), thereby resembling the time profile of the wild-type strain on d-glucose, albeit with lower ST concentrations. Thus, it appears that carbon assimilation rate and the resulting biomass production rate inversely correlate with ST formation in *A. nidulans* (Table 1), and slow growth is a prerequisite for mid-growth ST production to occur.

### 2.4. Sterigmatocystin Formation of A. nidulans in Chemostat-Type Continuous Cultures

The experiments analysed so far showed that ST-formation coincides with slower carbon utilization rate that in turn results in reduced growth rate. To address this hypothesis in more detail, a set of constant-mass, carbon-limited, chemostat-type continuous fermentations of the wild-type and the CreA-loss of function *A. nidulans* strain were performed at two different dilution rates at 0.090 h^−1^ and 0.020 h^−1^ with d-glucose as a carbon source. These two dilution rates (henceforth referred to as “high” and “low” growth rate) have previously been shown to represent a state of carbon catabolite repression (CCR) and derepression, respectively, in *A. nidulans* [22].

Cultures were grown batchwise for 24 h after inoculation. At the first 6–7 residence times of the cultivations, gradually attenuating oscillation of the specific biomass production occurred [23] after which the oscillation decreased to a non-significant level. The steady-state biomass concentration was 1.49 ± 0.20 g·L^−1^ irrespective of the dilution rate. The residual steady-state concentrations of d-glucose in the growth medium were 0.05–0.08 mM, which correlates well with the affinities of the high affinity hexose transporters of filamentous fungi [24], and prove that the cultures were indeed glucose-limited. Although the wild-type strain exhibited filamentous growth throughout and the CreA-negative strain exhibited some pellet formation (Supplementary Figure S1), these morphological differences were not considered relevant enough to affect the general experimental approach.

Analysis of ST production in these continuous cultures showed that the dilution rate (=specific growth rate) negatively correlates with ST production. At a high growth rate, no ST formed (Table 2). In contrast, low growth rate led to the formation of 0.5 mg·L^−1^ ST. Similar results were obtained with the CreA-loss of function mutant strain (Table 2), indicating that CreA does not regulate the formation of ST during growth on d-glucose.

## 3. Discussion

The experimental setup employed in this study allowed us to investigate the ST production of *A. nidulans* in liquid cultures, which—due to its more homogenous nature—are more suitable for quantitative kinetic studies than the agar-solidified medium widely used in labs interested in the mechanisms behind mycotoxin biosynthesis. Environmental factors are well known to influence ST formation [25,26,27], so we kept temperature, pH and DO levels at preset values. In *A. nidulans*, the effect of light on ST production was even shown to depend on the d-glucose concentration [28]. To further decrease the number of variables in our experimental system, all experiments were carried out in the dark. Under these conditions, independently of the carbon source available and in agreement with previous studies [17,18], *A. nidulans* produced much less ST in *veA1* background. In contrast, strains carrying the *veA* (wild-type) allele were capable of producing ST in both d-glucose and lactose-based minimal medium in a final concentration of up to 3 mg·L^−1^. This overall ST titer, while being far inferior to those attainable on plate cultures [29,30], still allowed us to safely determine the actual concentration of ST formed during fermentations.

Saprophytic fungi such as *A. nidulans* never encounter lactose in their natural habitats, which renders it a poor carbon substrate that results in slow growth (i.e., low specific growth rates). That condition will, however, facilitate the production of secondary metabolites—a term for pathways and products of metabolism that are believed not to be absolutely required for the survival of the organism—whose biosynthesis is often subject to regulation by the carbon source, generally through CreA/Cre1-dependent CCR [31,32,33,34]. Conversely, glucose-triggered CCR was described as being determined by the actual specific growth rate in *A. nidulans* [22], and also in the cellulase producer fungus *T. reesei* [35,36], as repression is overcome by growing fungi at or below certain defined, low growth rates. Interestingly, despite being established as a global metabolic regulator, little is known about the roles the CCR plays in mycotoxin biosynthesis [37]. Therefore, to the best of our knowledge, this is the first study that employed CreA mutants to directly address certain aspects of this point.

In considering the mechanisms of ST formation in batch cultures, one might suggest this to be initiated either by the reduced growth rate attainable on lactose or d-glucose after its exhaustion from the medium—or by the general relief from CCR. Since it was impossible to discriminate between these two effects in a batch or fed-batch culture, we turned to chemostat cultures where the specific growth rate was set according to the actual dilution rate. Essentially identical results to the wild-type reference strain were obtained with the CreA-loss of function mutant, providing evidence that CreA is not directly involved in the regulation of ST formation during growth on d-glucose. It should be noted, though, that lactose-grown batch cultures gave apparently contrasting results, as the onset of ST formation in the wild-type and the CreA-mutant cultures differed fundamentally. In the case of the former, ST appeared already at mid-growth, when lactose in the medium was still abundant, while in the latter, ST was not formed until the very late stationary phase, when lactose was long depleted. We speculate that this difference is caused by the enhanced lactose utilization rate of the CreA mutant (NB: basal level transcription of the lactose utilizing machinery in *A. nidulans* is repressed by CreA [38], and thus CreA-mutants are capable of faster growth on this carbon source than the wild-type strain). Similarly, d-glucose utilization rates were also reported to inversely correlate to AF production in *A. parasiticus* [39].

Since the residual d-glucose concentration was set close to zero in each chemostat culture irrespective of the dilution rate, we also concluded that the depletion of d-glucose itself is not an initiator—as one might argue upon inspecting the time-profiles of the batch and fed-batch cultures—but rather a prerequisite for ST biosynthesis through the cessation of growth. 

Finally, what does this study tell us about the relationship between lactose assimilation and ST formation? As opposed to CCR and carbon availability in the medium, it is the growth rate—the likely most fundamental parameter of a cell culture—that seems to have a direct, causal relationship with ST formation. Lactose is a poor carbon source for *A. nidulans* [38,40], and we believe the low growth rate caused by its slow assimilation will trigger sustained ST formation. By the same token, faster growth means the fungus has to put more resources into primary metabolism by creating cell materials needed for growth, and has less ability to shunt to ST. When lactose utilization rate gets higher (as in the CreA loss-of-function mutants), ST production will not occur before its complete exhaustion, just like on d-glucose. While we cannot yet link our observations to a defined molecular step, it is to be noted that in *Pichia pastoris*, carbon source-responsive genes and transcription factors are upregulated at low growth rates [41]. Recent evidence also suggests that fungal secondary metabolite gene clusters are differentially regulated by the various sugars available [34,42]. One might even consider the preservative nature of honey over AF contamination [43,44] to be related to the abundance of the rapidly assimilated d-fructose.

Another possible explanation for the lactose effect is that lactose catabolism—which is exclusively intracellular in *A. nidulans* [45]—may generate an endogenous inducer that is directly or indirectly able to stimulate ST biosynthesis in this fungus. We are currently testing this hypothesis.

## 4. Materials and Methods

### 4.1. Strains and Cultivation Conditions

*A. nidulans* strains used in this study are listed in Table 3. Conventional genetic techniques based on meiotic recombination were used to create a carbon catabolite derepressed *ΔcreA* strain with *veA+* background by Clutterbuck [46]. *A. nidulans* ZNEF 8.55—a *ΔcreA* mutant with a *veA+* (wild-type) allele—was obtained through crossing strains *creAΔ4* (carrying *ΔcreA* and *veA1* mutations) and RJMP 155.55 (carrying a *veA+* allele). Progenies were selected using a PCR-based method [47]. The primers used to differentiate between *veA* and the truncated *veA1* alleles employ the same reverse primer (5′-CTTGGCGCTGTAGACGATAA) and two different forward primers (5′-TGTGTTATCC CATCAAGAGG and 5′-TGTGTTATCCCATCAAGAGT) that anneals to the *veA+* or *veA1* sequence, respectively. The annealing and extension conditions of the PCR reactions were set at 56 °C for 30 s and 72 °C for 1 min, respectively [47]. The lactose utilization system of the progenies was tested by growing them on solid medium containing 1% d-glucose in the presence of X-gal (5-bromo-4-chloro-3-indoly1-β-d-galactoside) as described by Shroff et al. [48].

The modified *Aspergillus* Minimal Medium (AMM2) for shake flask cultures and submerged (liquid) bioreactor cultivations (referred to as ‘fermentations’) was formulated and inoculated as described by Fekete et al. [45]. AMM2 is similar to AMM [51] but includes ammonium dihydrogen phosphate (NH_4_H_2_PO_4_; 8 g·L^−1^) as the sole nitrogen source, as well as 0.1 g·L^−1^ calcium chloride (CaCl_2_). Vitamins and other supplements were added from sterile stock solutions.

To quantitatively assess specific growth rates, biomass and ST yields, fermentations were carried out in 9 L glass bioreactors, (Inel Ltd., Budapest, Hungary) with a working volume of 6 L and equipped with two six-blade Rushton disc turbine impellers. The pH was controlled with the automatic addition of 3 mol·L^−1^ H_3_PO_4_ or 3 mol·L^−1^ NaOH solutions. Dissolved oxygen (DO) levels were maintained at 20% saturation and were controlled by means of the agitation (=stirring) rate. To minimize medium loss, the waste gas was cooled in a reflux condenser connected to an external cooling bath (4 °C) before exiting the system. Carbon sources, unless stated differently, were used at 1.5% (*w*/*v*) initial concentration. Mycelia were pregrown in AMM2 containing glycerol as a carbon source, harvested after 24 h by filtration on a sintered glass funnel, washed with cold sterile tap water and transferred into fresh AMM2 containing other carbon sources. Shake-flask cultures were inoculated with 10^8^ *A. nidulans* conidia per 100 mL of medium and incubated at 37 °C in 500 mL glass Erlenmeyer flasks (VWR International Ltd., Debrecen, Hungary) in a rotary shaker (Infors AG, Bottmingen, Switzerland) at 200 rpm. To exclude any regulatory effects by light [47,52], fermentations were performed in constant darkness.

Fed-batch fermentations with repeated supplemental d-glucose feeding (10–10 g·L^−1^) were performed in the same 6 L bioreactor. The temperature, pH, agitation and aeration conditions were as for the batch fermentation.

Chemostat-type continuous cultivations (i.e., where the bioreactor is continuously fed with fresh growth medium while concurrently draining spent medium out at the same rate, thereby keeping the culture volume constant) were carried out in a 2.5 L glass bioreactor (Inel Ltd.) with a working volume of 2 L, equipped with one six-blade Rushton disc turbine impeller. The temperature and pH were as for the batch cultivations; light intrusion was similarly prevented. Agitation and aeration rates were set at 300 rpm and 0.3 vvm, respectively. The feeding AMM2 medium contained 3 g·L^−1^ d-glucose as a sole carbon source, a concentration low enough to make the culture carbon-limited. To avoid fungal wall growth, glass parts of the reactor were treated with the anti-adhesive agent Sigmacote^®^ prior cultivations. Polypropylene glycol 2000 (Union Carbide Chemicals & Plastics, Versoix, Switzerland) was used as antifoam agent; a few drops were injected into the bioreactor once a day through a membrane filter (Millipore Kft., Budapest, Hungary). The onset of steady state was established when no changes in the dry cell mass could be observed in three successive samples taken over a period of three residence times.

### 4.2. Analytical Methods

Mycelial dry weight (DCW) was determined from 20 mL culture aliquots. The biomass was harvested and washed on a preweighted glass wool filter by suction filtration and the filter dried at 80 °C until constant weight. Data were averaged and deviated by not more than 14%.

The actual concentration of d-glucose, glycerol and lactose was determined by HPLC analysis with a proton exchange column (Bio-Rad Aminex HPX-87H^+^; Bio-Rad Laboratories, Hercules, CA, USA) using isocratic elution with 10 mM H_2_SO_4_ at 55 °C and refractive index detection.

ST was first concentrated in an organic phase due to the relatively low (a few mg per L) level of this compound even in complex fermentation media. A 20 mL sample was extracted thrice with 10 mL of ethyl-acetate, followed by the organic phase being collected and evaporated. Samples were then re-suspended in 1 mL of aceto-nitrile. The isolation of the ST from solid media has a similar method as outlined above but before extraction the cylindrical shaped agar pieces cut directly off the plates had to be melted. The standard error of the extraction was below 5%. Reverse phase high performance liquid chromatography coupled to ultra-violet detection (RP-HPLC-UV; HP 1090 Series L/M Liquid Chromatographs, Agilent-Technologies, Waldbronn, Germany) and authentic standards have been employed to determine ST concentrations (see Supplementary Figure S2 for chromatograms and retention times). The mobile phase applied was water: acetonitrile (4: 6) mixture buffered with acetic-acid and Na-acetate, at a flow rate of 0.5 mL·min^−1^ with isocratic elution. Temperature of the column was kept at T = 55 °C, detection occurred at λ = 245nm. Since ST is a relatively stable molecule (pK_a_ = 9.58), pH of the mobile phase (pH 4.76) did not modify structure and thus elution properties, but the acidic solvent was necessary to separate ST from by-products of fungal metabolites. Under these conditions the retention time of ST is 11.9 min.

Fungal morphology was investigated by means of an Axio-Vision AC quantitative image analyser system (Version 4.6.3, Carl Zeiss, Oberkochen, Germany, 2007). Samples were analysed under a Zeiss AxioImager microscope (Carl Zeiss, Oberkochen, Germany), equipped with AxioCam MRc5 camera.

### 4.3. Genomic DNA Isolation

Samples of *A. nidulans* mycelia were collected and processed as described by Fekete et al. [38]. For nucleic acid isolation, frozen biomass was ground to dry powder using liquid nitrogen-chilled mortar and pestle. Genomic DNA was extracted using NucleoSpin Plant II (Macherey-Nagel, Düren, Germany). The concentration and purity of the DNA samples were determined by using a NanoDrop 2000 spectrophotometer (Thermo Fisher Scientific, Wilmington, DE, USA).

### 4.4. Reproducibility

All the data presented are means of 3–5 independent experiments (=biological replicates). The data were analyzed and visualized by SIGMAPLOT (Version 12.0, Jandel Scientific, San Jose, CA, USA, 2011), and standard deviations (SDs) were determined for each procedure. The SD values were always <14% of the mean values. For chemostat cultures, two to four separate steady-states (i.e., independently initiated and run continuous cultures at the constant-mass stage) were sampled and analysed for each dilution rate.

### 4.5. Chemicals

All chemicals were of analytical grade, and except where noted otherwise, were purchased from Sigma-Aldrich Ltd., Budapest, Hungary.

## Figures and Tables

**Table 1:** Derived kinetic parameters of *A. nidulans* cultures.

Strain, Carbon Source	Carbon Utilization Rate (g/h)	Biomass Production Rate (g_DCW_/h)
Wild-type, d-glucose	0.30 ± 0.03	0.15 ± 0.02
Wild-type, lactose	0.14 ± 0.02	0.07 ± 0.02
CreA mutant, d-glucose	0.29 ± 0.03	0.14 ± 0.01
CreA mutant, lactose	0.27 ± 0.03	0.13 ± 0.02

**Table 2:** Sterigmatocystin formation of an *A. nidulans* wild-type and a CreA loss-of-function mutant strain in chemostat-type continuous cultures as a function of the dilution rate.

Strain	Dilution Rate (1/h)	Steady-State Sterigmatocystin Concentration (mg/L)
Wild-type (*veA+*)	0.09	<0.1
	0.02	0.5 ± 0.07
CreA mutant (*veA+*)	0.09	<0.1
	0.02	0.4 ± 0.05

**Table 3:** *A. nidulans* strains used in this work.

Strain	Genotype	Reference
RDIT 9.32	***veA+***	Tsitsigiannis et al. [49]
(FGSC #A1252)		
RDIT 2.3	***veA1***	Tsitsigiannis et al. [49]
(FGSC #A1830)		
R21	*yA2*, *pabaA1*; ***veA1***	Fantes and Roberts [50]
(FGSC #A1228)		
V100 ^1^	***creAΔ4***; *pantoB100*; ***veA1***	Shroff et al. [48] ^2^
RJMP 155.55	*wA3*; *riboB2*, ***veA+***	Keller Lab (unpublished)
ZNEF 8.55 ^3^	***creAΔ4***; *wA3*; *pantoB100*; *riboB2*, ***veA+***	This study

^1^ Offspring from an outcross of the original mutant (Dr. Michel Flipphi, unpublished); ^2^ Reference to the original *creA****Δ***4 mutant strain; ^3^ Offspring of a cross between V100 and RJMP 155.55.

## Supplementary Materials

The following are available online at www.mdpi.com/2072-6651/8/12/354/s1, Figure S1: representative light microscopic image of mycelia from the four chemostat-type continuous cultures analysed in this study, Figure S2: RP-HPLC-UV chromatograms showing an ST standard, a sample and a standard addition procedure.

## References

[1] Rank C., Nielsen K.F., Larsen T.O., Varga J., Samson R.B.A., Frisvad J.S. (2011). Distribution of sterigmatocystin in filamentous fungi. Fungal Biol..

[2] Wilkinson H.H., Ramaswamy A., Sung C.S., Keller N.P. (2004). Increased conidiation associated with progression along the sterigmatocystin biosynthetic pathway. Mycology.

[3] Wogan G.N. (1992). Aflatoxins as risk for hepatocellular carcinoma in humans. Cancer Res..

[4] Hopwood D.A., Sherman D.H. (1990). Molecular genetics of polyketides and its comparison to fatty acid biosynthesis. Annu. Rev. Genet..

[5] Purchase I.F.H., van der Watt J.J. (1970). Carcinogenicity of sterigmatocystin. Food Cosmet. Toxicol..

[6] Barnes S.E., Dola T.P., Bennett J.W., Bhatnagar D. (1994). Synthesis of sterigmatocystin on a chemically defined medium by species of *Aspergillus* and *Chaetomium*. Mycopathologia.

[7] Amaike S., Keller N.P. (2011). *Aspergillus* *flavus*. Annu. Rev. Phytopathol..

[8] Bok J.W., Albright J.C., Ye R., Mead D., Wagner M., Krerowicz A., Goering A.W., Kelleher N.L., Keller N.P., Wu C.-C. (2015). Fungal artificial chromosomes for mining of the fungal secondary metabolome. BMC Genom..

[9] Hodges R.L., Hodges D.W., Goggans K., Xuei X., Skatrud P., McGilvray D. (1994). Genetic modification of an echinocandin B-producing strain of *Aspergillus nidulans* to produce mutants blocked in sterigmatocystin biosynthesis. J. Ind. Microbiol..

[10] Penalva N.A., Rowlands R.T., Turner G. (1998). The optimization of penicillin biosynthesis in fungi. Trends Biotechnol..

[11] Kennedy J., Auclair K., Kendrew S.G., Park C., Vederas J.C., Hutchinson C.R. (1999). Modulation of polyketide synthase activity by accessory proteins during lovastatin biosynthesis. Science.

[12] Hansen B.G., Mnich E., Nielsen K.F., Nielsen J.B., Nielsen M.T., Mortensen U.H., Larsen T.O., Patil K.R. (2012). Involvement of a natural fusion of a cytochrome P450 and a hydrolase in mycophenolic acid biosynthesis. Appl. Environ. Microbiol..

[13] Neely F.L., Emerson C.S. (1990). Determination of sterigmatocystin in fermentation broths by reversed-phase high-performance liquid chromatography using post-column fluorescence enhancement. J. Chromatogr..

[14] Hodges R.L., Kelkar H.S., Xuei X., Skatrud P.L., Keller N.P., Adams T.H., Kaiser R.E., Vinci V.A., McGilvray D. (2001). Characterization of an echinocandin B-producing strain blocked for sterigmatocystin biosynthesis reveals a translocation in the *stcW* gene of the aflatoxin biosynthetic pathway. J. Ind. Microbiol..

[15] Maggio-Hall L.A., Wilson R.A., Keller N.P. (2005). Fundamental contribution of β-oxidation to polyketide mycotoxin production in planta. Mol. Plant Microbe Interact..

[16] Tsitsigiannis D.I., Kowieski T.M., Zarnowski R., Keller N.P. (2005). Three putative oxylipin biosynthetic genes integrate sexual and asexual development in *Aspergillus nidulans*. Microbiology.

[17] Calvo A.M. (2008). The VeA regulatory system and its role in morphological and chemical development in fungi. Fungal Genet. Biol..

[18] Stinnett S.M., Espeso E.A., Cobeño L., Araújo-Bazán L., Calvo A.M. (2007). *Aspergillus nidulans* VeA subcellular localization is dependent on the importin α carrier and on light. Mol. Microbiol..

[19] Septien I., Blanco J.L., Suarez G., Cutuli M.T. (1994). Solubility and stability of sterigmatocystin in aqueous solution. Mycotoxin Res..

[20] Hamid A.B., Smith J.E. (1987). Degradation of aflatoxin by *Aspergillus flavus*. J. Gen. Microbiol..

[21] Wu Q., Jezkova A., Yuan Z., Pavlikova L., Dohnal V., Kuca K. (2009). Biological degradation of aflatoxins. Drug Metab. Rev..

[22] Ilyés H., Fekete E., Karaffa L., Fekete É., Sándor E., Szentirmai A., Kubicek C.P. (2004). CreA-mediated carbon catabolite repression of β-galactosidase formation in *Aspergillus nidulans* is growth rate dependent. FEMS Microbiol. Lett..

[23] Brown A., McNeil B., Harvey L.M. (1990). Fed-batch and continuous culture. Fermentation: A Practical Approach.

[24] Jorgensen T.R., van Kuyk P.A., Poulsen B.R., Ruijter G.J.G., Visser J., Iversen J.J.L. (2007). Glucose uptake and growth of glucose-limited chemostat cultures of Aspergills niger and a disruptant lacking MstA, a high-affinity glucose transporter. Microbiology.

[25] Abdollahi A., Buchanan R.L. (1981). Regulation of aflatoxin biosynthesis: Induction of aflatoxin production by various carbohydrates. J. Food Sci..

[26] Keller N.P., Nesbitt C., Sarr B., Philips T.D., Burow G.B. (1997). pH regulation of sterigmatocystin and aflatoxin biosynthesis in *Aspergillus* spp.. Phytopathology.

[27] Price M.S., Conners S.B., Tachdjian S., Kelly R.B., Payne G.A. (2005). Aflatoxin conducive and non-conducive growth conditions reveal new gene associations with aflatoxin production. Fungal Genet. Biol..

[28] Atoui A., Kastner C., Larey C.M., Thokala R., Etxebeste O., Espeso E.A., Fischer R., Calvo A.M. (2010). Cross-talk between light and glucose regulation controls toxin production and morphogenesis in *Aspergillus nidulans*. Fungal Genet. Biol..

[29] Calvo A.M., Wilson R.A., Bok J.W., Keller N.P. (2002). Relationship between secondary metabolism and fungal development. Microbiol. Mol. Biol. Rev..

[30] Hicks J.K., Yu J.H., Keller N.P., Adams T.H. (1997). *Aspergillus* sporulation and mycotoxin production both require inactivation of the FadA Gα protein-dependent signaling pathway. EMBO J..

[31] Aharonowitz Y., Cohen G., Martin J.F. (1992). Penicillin and cephalosporin biosynthetic genes: Structure, organization, regulation and evolution. Annu. Rev. Microbiol..

[32] Brakhage A.A. (1998). Molecular regulation of β-lactam biosynthesis in filamentous fungi. Microbiol. Mol. Biol. Rev..

[33] Espeso E.A., Peñalva M.A. (1992). Carbon catabolite repression can account for the temporal pattern of expression of a penicillin biosynthetic gene in *Aspergillus nidulans*. Mol. Microbiol..

[34] Zhang X., Zhu Y., Bao L., Gao L., Yao G., Li Y., Yang Z., Li Z., Zhong Y., Li F. (2016). Putative methyltransferase LaeA and transcription factor CreA are necessary for proper asexual development and controlling secondary metabolic gene cluster expression. Fungal Genet. Biol..

[35] Karaffa L., Fekete E., Gamauf C., Szentirmai A., Kubicek C.P., Seiboth B. (2006). d-galactose induces cellulase gene expression in *Hypocrea jecorina* at low growth rates. Microbiology.

[36] Portnoy T., Margeot A., Linke R., Atanasova L., Fekete E., Sándor E., Hartl L., Karaffa L., Druzhinina I.S., Seiboth B. (2011). The CRE1 carbon catabolite repressor of the fungus Trichoderma reesei: A master regulator of carbon assimilation. BMC Genom..

[37] Hicks J.K., Lockington R.A., Strauss J., Dieringer D., Kubicek C.P., Kelly J., Keller N.P. (2001). RcoA has pleiotropic effects on *Aspergillus nidulans* cellular development. Mol. Microbiol..

[38] Fekete E., Karaffa L., Seiboth B., Fekete É., Kubicek C.P., Flipphi M. (2012). Identification of a permease gene involved in lactose utilisation in *Aspergillus nidulans*. Fungal Genet. Biol..

[39] Shih C.N., Marth E.H. (1974). Some cultural conditions that control biosynthesis of lipid and aflatoxin by *Aspergillus parasiticus*. Appl. Microbiol..

[40] Fekete E., Orosz A., Kulcsár L., Kavalecz N., Flipphi M., Karaffa L. (2016). Characterization of a second physiologically relevant lactose permease gene (*lacpB*) in *Aspergillus nidulans*. Microbiology.

[41] Rebnegger C., Graf A.B., Valli M., Steiger M.G., Gasser B., Maurer M., Mattanovich D. (2014). In *Pichia pastoris*, growth rate regulates protein synthesis and secretion, mating and stress response. Biotechnol. J..

[42] Kumar D., Barad S., Chen Y., Luo X., Tannous J., Dubey A., Glam N., Tian S., Li B., Keller N.P. (2016). LaeA regulation of secondary metabolism modulates virulence in *Penicillium expansum* and is mediated by sucrose. Mol. Plant Pathol..

[43] Hanif M., Khattak M.K., Rehman M.U., Ramzan M., Amin M., Aamir M., Sher S.S., Hafizullah, Khan S., Saeed M. (2015). Effect of drying temperature and natural preservatives on reducing aflatoxins in solar dried persimmon (*Diospyros kaki* L). Proc. Pak. Acad. Sci..

[44] Wellford T.E.T., Eadie T., Llewellyn G.C. (1978). Evaluating, the inhibitory action of honey on fungal growth, sporulation, and aflatoxin production. Z. Lebensm. Unters. Forsch..

[45] Fekete E., Karaffa L., Sándor E., Seiboth B., Bíró S., Szentirmai A., Kubicek C.P. (2002). Regulation of the intracellular β-galactosidase activity of *Aspergillus nidulans*. Arch. Microbiol..

[46] Clutterbuck A.J., King R.C. (1974). *Aspergillus* *nidulans*. Handbook of Genetics, Bacteria, Bacteriophages, and Fungi.

[47] Han K.H., Park J.S., Chae K.S., Han D.M. (2010). Simple identification of *veA1* mutation in *Aspergillus nidulans*. J. Microbiol..

[48] Shroff R.A., O’Connor S.M., Hynes M.J., Lockington R.A., Kelly J.M. (1997). Null alleles of *creA*, the regulator of carbon catabolite repression in *Aspergillus nidulans*. Fungal Genet. Biol..

[49] Tsitsigiannis D.I., Zarnowski R., Keller N.P. (2004). The lipid body protein, PpoA, coordinates sexual and asexual sporulation in *Aspergillus nidulans*. J. Biol. Chem..

[50] Fantes P.A., Roberts C.F. (1973). β-Galactosidase activity and lactose utilization in *Aspergillus nidulans*. J. Gen. Microbiol..

[51] Pontecorvo G., Roper J.A., Chemmons L.M., Macdonald K.D., Bufton A.W.J. (1953). The genetics of *Aspergillus nidulans*. Adv. Genet..

[52] Bayram Ö., Krappmann S., Ni M., Bok J.W., Helmstaedt K., Valerius O., Braus-Stromeyer S., Kwon N.J., Keller N.P., Yu J.H. (2008). VelB/VeA/LaeA complex coordinates light signal with fungal development and secondary metabolism. Science.

