# Supplementary material for: Growth-Phase Sterigmatocystin Formation on Lactose Is Mediated via Low Specific Growth Rates in Aspergillus nidulans
Source: Toxins (Basel). 2016 Nov 28;8(12):354. doi: 10.3390/toxins8120354 (PMC5198170; doi:10.3390/toxins8120354)
Supplement: Supplementary file 1 [file toxins-08-00354-s001.pdf]

Supplementary Materials: Growth-Phase Sterigmatocystin Formation on Lactose Is Mediated via Low Specific Growth Rates in *Aspergillus nidulans*

Zoltán Németh, Ákos P. Molnár, Balázs Fejes, Levente Novák, Levente Karaffa, Nancy P. Keller and Erzsébet Fekete

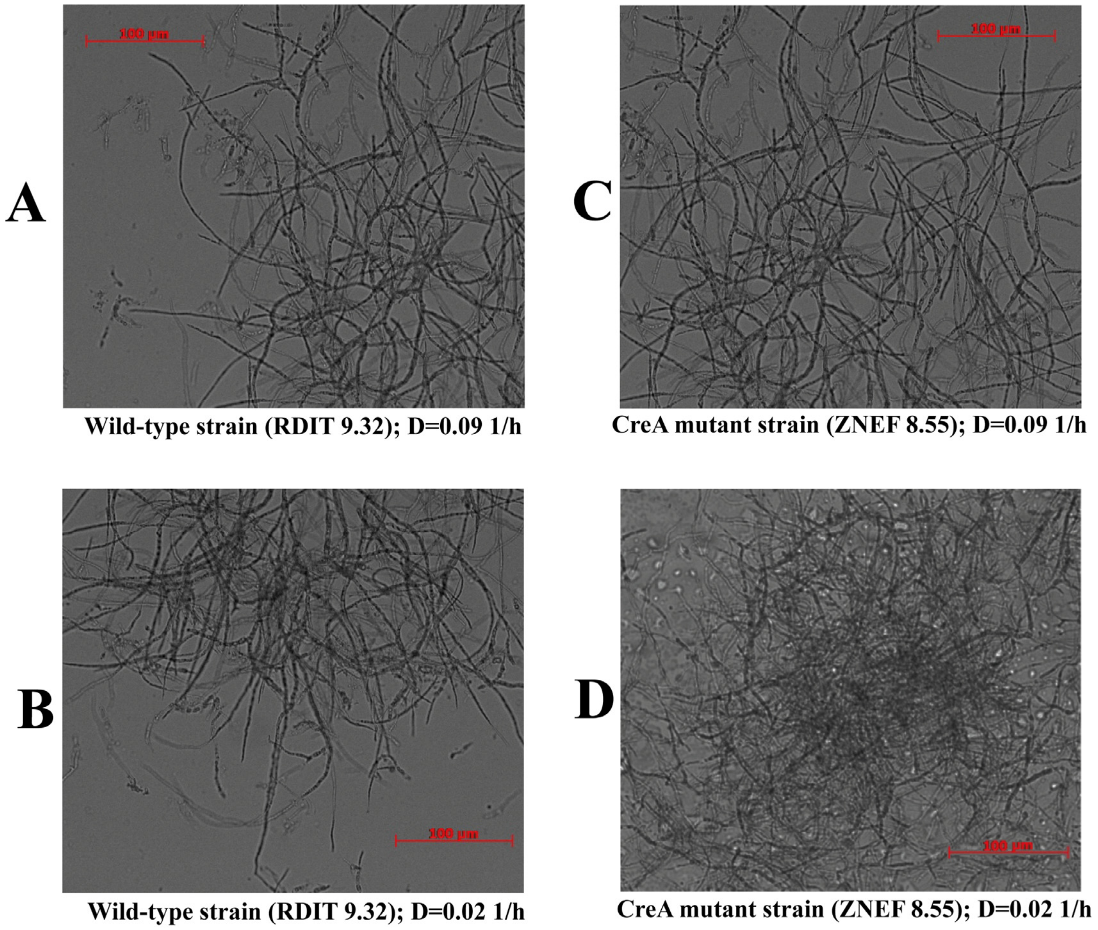

Figure S1. Representative light microscopic image of mycelia from the four chemostat-type continuous cultures analysed in this study.

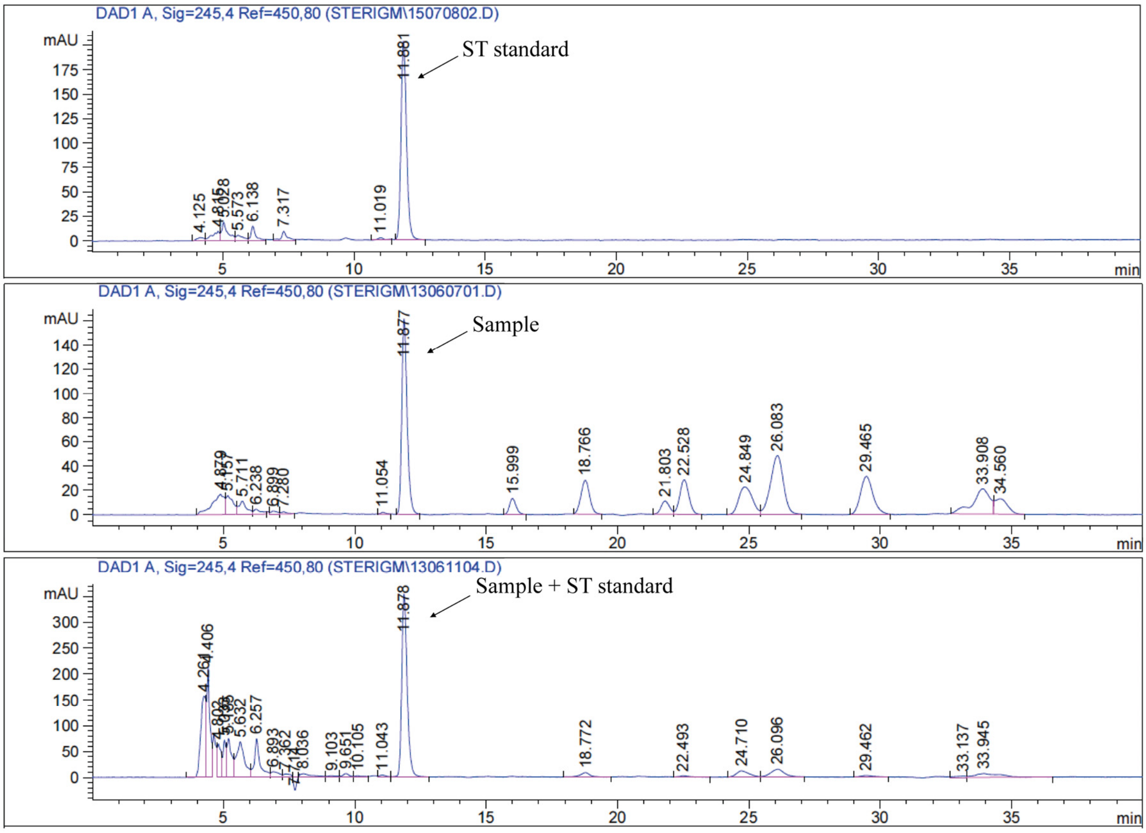

Figure S2. RP-HPLC-UV chromatograms showing an ST standard, a sample and a standard addition procedure.
